# Population‐based study of long‐term functional outcomes after prostate cancer treatment

**DOI:** 10.1111/bju.13179

**Published:** 2015-06-23

**Authors:** Sigrid Carlsson, Linda Drevin, Stacy Loeb, Anders Widmark, Ingela Franck Lissbrant, David Robinson, Eva Johansson, Pär Stattin, Per Fransson

**Affiliations:** ^1^ Department of Surgery, Urology Service Memorial Sloan‐Kettering Cancer Center New York NY USA; ^2^ Department of Urology Sahlgrenska Academy at Göteborg University Göteborg Sweden; ^3^ Regional Cancer Center Uppsala University Hospital Uppsala Sweden; ^4^ New York University and Manhattan Veterans Affairs Medical Center New York NY USA; ^5^ Department of Radiation Sciences, Oncology Umeå University Umeå Sweden; ^6^ Department of Oncology Sahlgrenska Academy at Göteborg University Göteborg Sweden; ^7^ Department of Surgery and Perioperative Sciences, Urology and Andrology Umeå University Hospital Umeå Sweden; ^8^ Department of Urology Ryhov County Hospital Jönköping Sweden; ^9^ Department of Surgical Sciences University Hospital of Uppsala Uppsala Sweden; ^10^ Department of Nursing Umeå University Umeå Sweden

**Keywords:** prostate cancer, erectile dysfunction, urinary incontinence, prostatic neoplasms/therapy, quality of life

## Abstract

**Objective:**

To evaluate long‐term urinary, sexual and bowel functional outcomes after prostate cancer treatment at a median (interquartile range) follow‐up of 12 (11–13) years.

**Patients and Methods:**

In this nationwide, population‐based study, we identified 6 003 men diagnosed with localized prostate cancer (clinical local stage T1–2, any Gleason score, prostate‐specific antigen <20 ng/mL, NX or N0, MX or M0) between 1997 and 2002 from the National Prostate Cancer Register, Sweden. The men were aged ≤70 years at diagnosis. A control group of 1 000 men without prostate cancer were also selected, matched for age and county of residence. Functional outcomes were evaluated with a validated self‐reported questionnaire.

**Results:**

Responses were obtained from 3 937/6 003 cases (66%) and 459/1 000 (46%) controls. At 12 years after diagnosis and at a median age of 75 years, the proportion of cases with adverse symptoms was 87% for erectile dysfunction/sexual inactivity, 20% for urinary incontinence and 14% for bowel disturbances. The corresponding proportions for controls were 62, 6 and 7%, respectively. Men with prostate cancer, except those on surveillance, had an increased risk of erectile dysfunction compared with the men in the control group. Radical prostatectomy was associated with an increased risk of urinary incontinence (odds ratio [OR] 1.89, 95% confidence interval [CI] 1.36–2.62) and radiotherapy increased the risk of bowel dysfunction (OR 2.46, 95% CI 1.73–3.49) compared with men in the control group. Multi‐modal treatment, in particular treatment including androgen deprivation therapy (ADT), was associated with the highest risk of adverse effects; for instance, radical prostatectomy followed by radiotherapy and ADT was associated with an OR of 3.74 (95% CI 1.76–7.95) for erectile dysfunction and an OR of 3.22 (95% CI 1.93–5.37) for urinary incontinence.

**Conclusion:**

The proportion of men who experienced a long‐term impact on functional outcomes after prostate cancer treatment was substantial.

## Introduction

Erectile dysfunction, urinary incontinence and bowel dysfunction after prostate cancer treatment have been shown to negatively affect quality of life in the short and intermediate term 1. Two years after primary treatment for prostate cancer, overall sexual health issues caused moderate or severe distress in 43% of men after radical prostatectomy and in 30–37% of men after radiotherapy. The corresponding figures for urinary symptoms were 7 and 11–16%, respectively. Bowel dysfunction caused distress in 11% of men after radiotherapy 1.

In the USA, ~75% of men with localized prostate cancer are treated with radical prostatectomy or radiotherapy 2. In Sweden, the proportion of men who receive curative treatment is fairly similar for men with intermediate‐risk disease, whereas active surveillance is more commonly used among Swedish men with very‐low‐risk (59%) and low‐risk (41%) prostate cancer as compared with the USA 3, 4. Androgen deprivation therapy (ADT) is the primary treatment in 14% of men with localized prostate cancer (mostly intermediate and high risk) in the USA 2 and 10% in Sweden 3.

Men with localized low‐ and intermediate‐risk prostate cancer have a long life expectancy with prostate cancer‐specific mortality rates of 3–4% at 10 years after curative treatment or surveillance 5 and 2–10% at 15 years after radical prostatectomy 6. There is therefore a need for more knowledge regarding the long‐term implications of prostate cancer treatment on urinary, sexual and bowel function. In the US Prostate Cancer Outcomes Study, erectile dysfunction or sexual inactivity was almost universal 15 years after treatment, with rates of 87% after radical prostatectomy and 94% after radiotherapy. The prevalence of urinary incontinence was 18 and 9%, and for bowel urgency was 22 and 36%, respectively 7.

A limitation of previous studies is that they mainly included men who underwent primary treatment with curative intent 1, 7. These studies did not include a comparison group and did not address the functional outcomes after secondary therapy other than ADT. A recent study from the US Cancer of the Prostate Strategic Research Endeavor (CaPSURE) registry, which covers men with localized prostate cancer from 45 community‐based urology practices, diagnosed between 1995 and 2011, prospectively evaluated functional outcomes up to 10 years after various prostate cancer treatments, including secondary treatments. The study showed significant declines in health‐related quality of life over time, adjusting for age, year of treatment, comorbidities, health insurance status, cancer progression risk at diagnosis, Cancer of the Prostate Risk Assessment (or CAPRA) risk score and secondary treatment. Surgery was found to have the greatest impact on sexual and urinary function, radiation had the greatest impact on bowel function and ADT on physical function; however, that study did not include a non‐prostate cancer control group, and therefore was unable to assess whether declines in functional outcomes were different from those in men in a similar age group who were free of disease 8.

Because of the scarcity of large‐scale population‐based studies reporting long‐term patient‐reported functional outcomes after prostate cancer treatment, our aim was to evaluate these outcomes for men from the National Prostate Cancer Register of Sweden (NPCR). We examined long‐term urinary, sexual and bowel function after conservative management, prostatectomy, radiation therapy, ADT and combinations thereof, and compared these results with those in a control group of men free of prostate cancer.

## Subjects and Methods

### Subjects

Since 1998, prostate cancer cases have been reported to the NPCR. Compared with the Swedish Cancer Registry, to which reporting is mandatory by law, the capture rate is 98% 9. The NPCR includes information on TNM stage, Gleason score, PSA levels at diagnosis and primary treatment. A detailed description of treatment patterns in the NPCR, such as radiation doses, planned nerve‐sparing surgery and type of ADT is available on the NPCR website 3.

The NPCR Follow‐Up Study was performed to assess long‐term prostate cancer treatment outcomes in men aged ≤ 70 years at date of diagnosis, who were diagnosed with localized prostate cancer clinical stage T1 or T2, any Gleason score, PSA < 20 ng/mL and no signs of lymph node metastases (N0 or NX) or bone metastases (M0 or MX) in 1997–2002. This study has been described in detail previously 5, 9. Using the unique Swedish personal identification number, we performed registry linkages to obtain information on comorbidities from the National Patient Register and on socio‐economic status from the longitudinal database of socio‐economic factors 10.

In September 2011, we identified 6 003 men from the NPCR Follow‐Up Study who were alive. We selected 1 000 prostate cancer‐free men matched on age (±1 year) and county of residence in the Swedish population in order to create a study‐specific comparison group. Linkage with the Swedish Cancer Registry was performed to ensure that the control subjects were free from prostate cancer. No data on comorbidities or socio‐economic status were available for control subjects and these important confounders could therefore not be adjusted for in any analysis. Instead, a secondary analysis restricted to men with prostate cancer only was performed, adjusting for age, comorbidities, marital status and education, with men on surveillance as the reference group, which is believed to be most similar with respect to functional outcomes as a control group.

### Outcome Assessment

We assessed the cumulative effect on functional outcomes after various prostate cancer treatments after a median (interquartile range [IQR]) time of 12 (11–13) years after diagnosis. The survey was carried out at varying times after prostate cancer treatment, providing a comprehensive cross‐sectional picture of the prevalence of long‐term functional outcomes in men with prostate cancer.

Functional outcomes were evaluated using a patient self‐reported questionnaire. Study participants were first asked to complete a web‐version of the questionnaire and if no response was received within 1 month, a paper questionnaire was sent by ordinary mail.

This questionnaire was based on the Prostate‐Cancer Symptom Scale self‐assessment, formerly known as the Questionnaire Umeå Fransson Widmark 1994, which was developed and validated by two of the authors 11. It includes a question regarding urinary incontinence assessing the use of pads, as well as validated questions regarding bowel function 12, health‐related quality of life 13, the International Index of Erectile Function‐5 (IIEF‐5) 14 and the IPSS 15.

The main endpoints were: urinary incontinence; erectile dysfunction or sexual inactivity; and bowel dysfunction. Secondary endpoints were urinary urgency and health‐related quality of life.

We used a composite outcome to assess sexual dysfunction defined as erectile dysfunction (an IIEF‐5 score ≤17 and/or reported use of alprostadil) or sexual inactivity. The use of phosphodiesterase type 5 inhibitors was not used for classification. The threshold score of 17 has previously been used 16, and follows the classification suggested by Rosen et al. 14, who developed the questionnaire for distinguishing between severe to moderate (scores 5–16) and mild (scores 17–21) and no erectile dysfunction (scores 22–25). In addition, we considered an IIEF‐5 score of 17 to be an appropriate threshold for the older age group under study, as opposed to the higher threshold of 21.

Urinary continence was defined as no leakage or occasional use of a protective pad, and urinary incontinence was defined as regular use of pads or diapers.

Bowel dysfunction was assessed through three validated questions inquiring about blood in stools, mucus discharge and faecal incontinence, each on a modified linear analogue scale. Bowel problems were then dichotomized and defined as no problems vs problems (‘a little’, ‘quite a bit’ or ‘much’).

### Ethics

The Research Ethics Board at Gothenburg University Hospital and Umeå University Hospital approved the study.

### Statistical Analysis

To investigate the association between treatment modality and functional outcome, logistic regression analysis adjusting for age was applied and 95% CIs were calculated around point estimates. A descriptive analysis of non‐responders was performed. To minimize any bias associated with conducting a complete case analysis only, missing values in the four outcome variables (erectile dysfunction, urgency, urinary incontinence and bowel dysfunction), were imputed, both for non‐responders to the questionnaire as well as for responders with missing values, both for cases and controls, using multivariate imputation by chained equations following the method described by van Buuren 17. Two alternative methods of imputing missing outcome values were used: imputation based on age only (primary) and imputation based on the variables age, comorbidity, marital status and education, excluding the men without prostate cancer.

Separate analyses were performed restricted to treated patients with prostate cancer, adjusting for age, comorbidity status, marital status and education. The statistical analyses were conducted using the R statistical program package, version 2.15.1 (R Foundation for Statistical Computing, Vienna, Austria).

## Results

**Figure 1:**
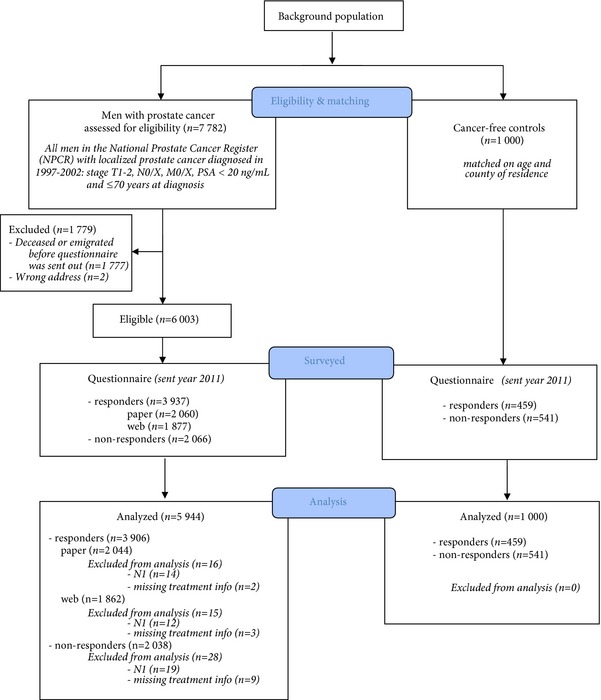
Flow chart of study participants.

Functional outcomes were assessed at a median (IQR) time of 12 (11–13) years after diagnosis. Responses to the survey were obtained from 3 937/6 003 cases (66%) and 459/1 000 (46%) controls (Fig. 1).

At the date of survey, the median (IQR) age was 75 (70–79) years for cases and 74 (70–78) years for controls. Men who had undergone radical prostatectomy as primary treatment were younger (median age 74 years at follow‐up) and had fewer comorbidities than men who received radiotherapy (median age 76 years) or primary ADT (median age 80 years). There were no material differences in socio‐economic status, education or marital status between men undergoing different treatments (Table 1).

**Table 1:** Patient characteristics by treatment

	Surveillance (*n *=* *670)	RP (*n *=* *2 640)	RT (*n *=* *857)	RP + RT (*n *=* *281)	ADT (*n *=* *394)	RP + ADT (*n *=* *357)	RT + ADT (*n *=* *549)	RT + RT + ADT (*n *=* *196)	All (*n *=* *5 944)
Median (IQR) age at diagnosis, years	65 (61–68)	61 (57–65)	64 (60–67)	61 (57–65)	68 (65–69)	63 (59–66)	65 (60–67)	61 (57–65)	63 (59–67)
Median (IQR) age at follow‐up, years	78 (74–81)	74 (69–78)	76 (72–80)	73 (69–77)	80 (77–82)	75 (71–79)	77 (73–80)	73 (69–77)	75 (71–79)
Age group at follow‐up, *n* (%)									
<70 years	69 (10.3)	671 (25.4)	128 (14.9)	82 (29.2)	10 (2.5)	74 (20.7)	68 (12.4)	52 (26.5)	1 154 (19.4)
70–74 years	114 (17.0)	799 (30.3)	213 (24.9)	83 (29.5)	31 (7.9)	75 (21.0)	135 (24.6)	61 (31.1)	1 511 (25.4)
≥75 years	487 (72.7)	1 170 (44.3)	516 (60.2)	116 (41.3)	353 (89.6)	208 (58.3)	346 (63.0)	83 (42.3)	3 279 (55.2)
Year of diagnosis, *n* (%)									
1997	77 (11.5)	169 (6.4)	39 (4.6)	12 (4.3)	30 (7.6)	23 (6.4)	29 (5.3)	6 (3.1)	385 (6.5)
1998	92 (13.7)	277 (10.5)	76 (8.9)	21 (7.5)	49 (12.4)	50 (14.0)	55 (10.0)	13 (6.6)	633 (10.6)
1999	96 (14.3)	387 (14.7)	133 (15.5)	25 (8.9)	60 (15.2)	78 (21.8)	89 (16.2)	20 (10.2)	888 (14.9)
2000	108 (16.1)	498 (18.9)	182 (21.2)	62 (22.1)	90 (22.8)	68 (19.0)	94 (17.1)	26 (13.3)	1 128 (19.0)
2001	145 (21.6)	571 (21.6)	225 (26.3)	75 (26.7)	74 (18.8)	65 (18.2)	132 (24.0)	57 (29.1)	1 344 (22.6)
2002	152 (22.7)	738 (28.0)	202 (23.6)	86 (30.6)	91 (23.1)	73 (20.4)	150 (27.3)	74 (37.8)	1 566 (26.3)
Socio‐economic status, *n* (%)									
Low	318 (47.5)	1 127 (42.7)	384 (44.8)	127 (45.2)	205 (52.0)	160 (44.8)	267 (48.6)	86 (43.9)	2 674 (45.0)
High	345 (51.5)	1 489 (56.4)	468 (54.6)	154 (54.8)	185 (47.0)	195 (54.6)	277 (50.5)	110 (56.1)	3 223 (54.2)
Other	7 (1.0)	24 (0.9)	5 (0.6)	0 (0.0)	4 (1.0)	2 (0.6)	5 (0.9)	0 (0.0)	47 (0.8)
Charlson comorbidity index, *n* (%)									
0	495 (73.9)	2 239 (84.8)	664 (77.5)	243 (86.5)	280 (71.1)	301 (84.3)	432 (78.7)	160 (81.6)	4 814 (81.0)
1	112 (16.7)	273 (10.3)	121 (14.1)	29 (10.3)	65 (16.5)	44 (12.3)	78 (14.2)	21 (10.7)	743 (12.5)
2	45 (6.7)	106 (4.0)	49 (5.7)	7 (2.5)	32 (8.1)	10 (2.8)	29 (5.3)	13 (6.6)	291 (4.9)
3+	18 (2.7)	22 (0.8)	23 (2.7)	2 (0.7)	17 (4.3)	2 (0.6)	10 (1.8)	2 (1.0)	96 (1.6)
Education, *n* (%)									
Low	257 (38.4)	829 (31.4)	308 (35.9)	87 (31.0)	180 (45.7)	121 (33.9)	210 (38.3)	68 (34.7)	2 060 (34.7)
Middle	259 (38.7)	1 065 (40.3)	328 (38.3)	122 (43.4)	140 (35.5)	145 (40.6)	210 (38.3)	82 (41.8)	2 351 (39.6)
High	152 (22.7)	733 (27.8)	218 (25.4)	72 (25.6)	73 (18.5)	89 (24.9)	129 (23.5)	46 (23.5)	1 512 (25.4)
Missing	2 (0.3)	13 (0.5)	3 (0.4)	0 (0.0)	1 (0.3)	2 (0.6)	0 (0.0)	0 (0.0)	21 (0.4)
Marital status, *n* (%)									
Married	476 (71.0)	2 075 (78.6)	634 (74.0)	223 (79.4)	296 (75.1)	273 (76.5)	424 (77.2)	151 (77.0)	4 552 (76.6)
Divorced	102 (15.2)	298 (11.3)	132 (15.4)	29 (10.3)	47 (11.9)	47 (13.2)	71 (12.9)	30 (15.3)	756 (12.7)
Widower	35 (5.2)	69 (2.6)	28 (3.3)	9 (3.2)	17 (4.3)	12 (3.4)	22 (4.0)	5 (2.6)	197 (3.3)
Never married	57 (8.5)	198 (7.5)	63 (7.4)	20 (7.1)	34 (8.6)	25 (7.0)	32 (5.8)	10 (5.1)	439 (7.4)
Risk group^a^, *n* (%)									
Low risk	526 (78.5)	1 625 (61.6)	478 (55.8)	123 (43.8)	171 (43.4)	125 (35.0)	182 (33.2)	70 (35.7)	3 300 (55.5)
Intermediate risk	138 (20.6)	947 (35.9)	351 (41.0)	146 (52.0)	210 (53.3)	193 (54.1)	312 (56.8)	104 (53.1)	2 401 (40.4)
High risk	6 (0.9)	68 (2.6)	28 (3.3)	12 (4.3)	13 (3.3)	39 (10.9)	55 (10.0)	22 (11.2)	243 (4.1)

RP, radical prostatectomy; RT, radiotherapy; ADT, androgen deprivation therapy.

a Risk group at diagnosis defined as: low risk; T1–2 and Gleason score ≤ 6 and PSA < 10 ng/mL; intermediate risk: T1‐2 and Gleason score 7 and/or PSA 10–20 ng/mL, high risk; T3, and/or Gleason score 8‐10 and/or PSA 20–50 ng/mL. Patients with regionally metastatic disease (N1 and/or PSA 50–100 ng/mL and no distant metastases, M0 or MX) and patients with distant metastasis (M1 and/or PSA ≥ 100 ng/mL) were not eligible. There were no missing data for risk group.

A substantial proportion of men (23%, 1 383/5 944) received secondary treatment at varying time points after primary treatment. A higher proportion of men with high‐risk cancer (53%, 128/243) underwent multiple treatments compared with men with low‐risk cancer (15%, 500/3 300), whereas surveillance was more often used in men with low‐risk cancer (16%, 526/3 300) than in men with high‐risk cancer (2%, 6/243; Table 1).

At follow‐up, a substantial proportion of men who had received any curative treatment or ADT experienced a negative impact on sexual, urinary and bowel function. In all, 87% of the men had erectile dysfunction or were sexually inactive, 20% reported urinary incontinence and 14% had bowel symptoms. The corresponding proportions for the men in the control group were 62, 6 and 7%, respectively.

### Sexual and Urinary Dysfunction

**Figure 2:**
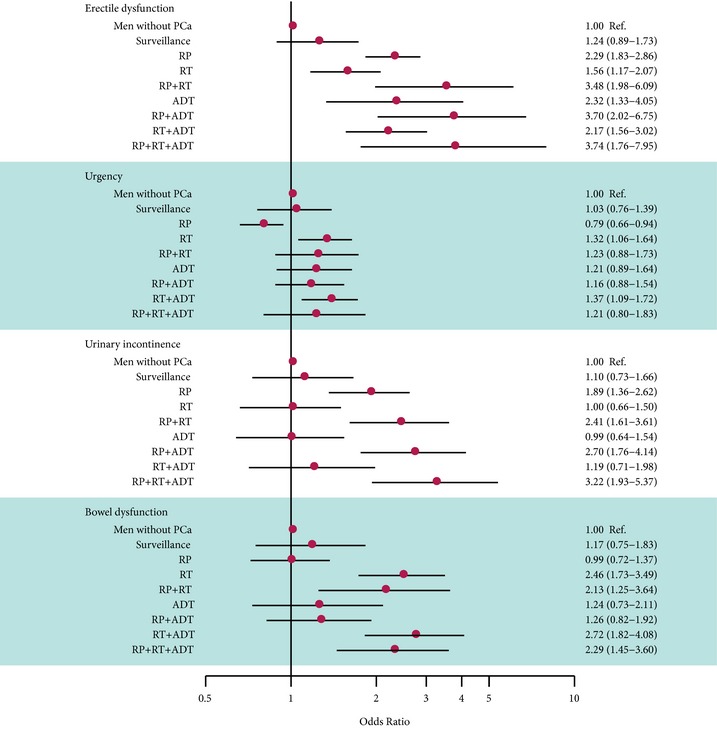
Odds ratios and 95% confidence intervals for the risk of adverse functional outcomes by treatment compared to controls and adjusted for age.

Compared with the men in the control group, the men treated for prostate cancer had an increased risk of erectile dysfunction. There was no statistically significant difference in men with erectile dysfunction among men on surveillance compared with the men in the control group: odds ratio (OR) 1.24 (95% CI 0.89–1.73). The risk was higher for men who had undergone radical prostatectomy (OR 2.29 [95% CI 1.83–2.86]) and single‐mode radiotherapy (OR 1.56 [95% CI 1.17–2.07]; Fig. 2). The risk of urinary incontinence was higher for men who underwent radical prostatectomy (OR 1.89 [95% CI 1.36–2.62]), and men who had prostatectomy followed by radiotherapy and ADT had a threefold higher risk of incontinence compared with the control group (OR 3.22 [95% CI 1.93–5.37]) (Fig. 2).

The risk of urinary urgency symptoms was higher after radiotherapy (OR 1.32 [95% CI 1.06–1.64]), but lower after radical prostatectomy (OR 0.79 [95% CI 0.66–0.94]) (Fig. 2).

Occurrence of erectile dysfunction, sexual inactivity and urinary incontinence after treatment was more common with increasing age at the time of the survey (Tables 2 and 3).

**Table 2:** Erectile function at survey, stratified by treatment and age group

	No erectile dysfunction *n* (%)		Erectile dysfunction^a^ *n* (%)		Not sexually active *n* (%)		Missing *n* (%)	
All age groups, *n* (%)								
Men without prostate cancer	160	(34.9)	57	(12.4)	200	(43.6)	42	(9.2)
Surveillance	69	(20.1)	62	(18.1)	159	(46.4)	53	(15.5)
RP	211	(11.4)	261	(14.0)	1 151	(61.9)	236	(12.7)
RT	87	(15.9)	93	(17.0)	297	(54.4)	69	(12.6)
RP + RT	15	(7.7)	27	(13.8)	138	(70.8)	15	(7.7)
ADT	12	(5.5)	19	(8.8)	138	(63.6)	48	(22.1)
RP + ADT	11	(4.6)	16	(6.6)	188	(78.0)	26	(10.8)
RT + ADT	35	(9.5)	54	(14.6)	225	(61.0)	55	(14.9)
RP + RT + ADT	6	(4.4)	14	(10.3)	100	(73.5)	16	(11.8)
Age < 70 years								
Men without prostate cancer	60	(60.6)	11	(11.1)	22	(22.2)	6	(6.1)
Surveillance	10	(25.0)	14	(35.0)	9	(22.5)	7	(17.5)
RP	111	(22.7)	94	(19.2)	225	(45.9)	60	(12.2)
RT	27	(31.8)	22	(25.9)	27	(31.8)	9	(10.6)
RP + RT	6	(10.3)	10	(17.2)	40	(69.0)	2	(3.4)
ADT	2	(33.3)	1	(16.7)	3	(50.0)	0	(0.0)
RP + ADT	5	(10.0)	4	(8.0)	36	(72.0)	5	(10.0)
RT + ADT	8	(16.7)	11	(22.9)	23	(47.9)	6	(12.5)
RP + RT + ADT	3	(8.3)	6	(16.7)	25	(69.4)	2	(5.6)
Age 70–74 years								
Men without prostate cancer	54	(37.8)	16	(11.2)	59	(41.3)	14	(9.8)
Surveillance	18	(27.7)	10	(15.4)	29	(44.6)	8	(12.3)
RP	57	(9.9)	95	(16.6)	358	(62.5)	63	(11.0)
RT	25	(18.2)	25	(18.2)	72	(52.6)	15	(10.9)
RP + RT	3	(5.6)	7	(13.0)	38	(70.4)	6	(11.1)
ADT	3	(11.5)	2	(7.7)	16	(61.5)	5	(19.2)
RP + ADT	2	(3.3)	4	(6.6)	49	(80.3)	6	(9.8)
RT + ADT	16	(17.2)	16	(17.2)	52	(55.9)	9	(9.7)
RP + RT + ADT	2	(4.1)	5	(10.2)	35	(71.4)	7	(14.3)
Age ≥75 years								
Men without prostate cancer	46	(21.2)	30	(13.8)	119	(54.8)	22	(10.1)
Surveillance	41	(17.2)	38	(16.0)	121	(50.8)	38	(16.0)
RP	43	(5.4)	72	(9.0)	568	(71.4)	113	(14.2)
RT	35	(10.8)	46	(14.2)	198	(61.1)	45	(13.9)
RP + RT	6	(7.2)	10	(12.0)	60	(72.3)	7	(8.4)
ADT	7	(3.8)	16	(8.6)	119	(64.3)	43	(23.2)
RP + ADT	4	(3.1)	8	(6.2)	103	(79.2)	15	(11.5)
RT + ADT	11	(4.8)	27	(11.8)	150	(65.8)	40	(17.5)
RP + RT + ADT	1	(2.0)	3	(5.9)	40	(78.4)	7	(13.7)

RP, radical prostatectomy; RT, radiotherapy; ADT, androgen deprivation therapy.

a International Index of Erectile Function‐5 score ≤ 17 or alprostadil use.

**Table 3:** Urinary continence at survey, stratified by treatment and age group

	Continent *n* (%)		Incontinent *n* (%)		Missing *n* (%)	
All age groups						
Men without prostate cancer	409	(89.1)	24	(5.2)	26	(5.7)
Surveillance	276	(80.5)	28	(8.2)	39	(11.4)
RP	1 283	(69.0)	387	(20.8)	189	(10.2)
RT	445	(81.5)	50	(9.2)	51	(9.3)
RP + RT	130	(66.7)	53	(27.2)	12	(6.2)
ADT	160	(73.7)	26	(12.0)	31	(14.3)
RP + ADT	139	(57.7)	78	(32.4)	24	(10.0)
RT + ADT	289	(78.3)	46	(12.5)	34	(9.2)
RP + RT + ADT	84	(61.8)	45	(33.1)	7	(5.1)
Age < 70 years						
Men without prostate cancer	93	(93.9)	2	(2.0)	4	(4.0)
Surveillance	31	(77.5)	4	(10.0)	5	(12.5)
RP	375	(76.5)	66	(13.5)	49	(10.0)
RT	75	(88.2)	4	(4.7)	6	(7.1)
RP + RT	44	(75.9)	12	(20.7)	2	(3.4)
ADT	5	(83.3)	1	(16.7)	0	(0.0)
RP + ADT	32	(64.0)	11	(22.0)	7	(14.0)
RT + ADT	43	(89.6)	2	(4.2)	3	(6.2)
RP + RT + ADT	27	(75.0)	8	(22.2)	1	(2.8)
Age 70–74 years						
Men without prostate cancer	124	(86.7)	9	(6.3)	10	(7.0)
Surveillance	51	(78.5)	8	(12.3)	6	(9.2)
RP	395	(68.9)	120	(20.9)	58	(10.1)
RT	117	(85.4)	9	(6.6)	11	(8.0)
RP + RT	36	(66.7)	13	(24.1)	5	(9.3)
ADT	20	(76.9)	2	(7.7)	4	(15.4)
RP + ADT	36	(59.0)	18	(29.5)	7	(11.5)
RT + ADT	77	(82.8)	10	(10.8)	6	(6.5)
RP + RT + ADT	31	(63.3)	14	(28.6)	4	(8.2)
Age ≥75 years						
Men without prostate cancer	192	(88.5)	13	(6.0)	12	(5.5)
Surveillance	194	(81.5)	16	(6.7)	28	(11.8)
RP	513	(64.4)	201	(25.3)	82	(10.3)
RT	253	(78.1)	37	(11.4)	34	(10.5)
RP + RT	50	(60.2)	28	(33.7)	5	(6.0)
ADT	135	(73.0)	23	(12.4)	27	(14.6)
RP + ADT	71	(54.6)	49	(37.7)	10	(7.7)
RT + ADT	169	(74.1)	34	(14.9)	25	(11.0)
RP + RT + ADT	26	(51.0)	23	(45.1)	2	(3.9)

RP, radical prostatectomy; RT, radiotherapy; ADT, androgen deprivation therapy.

### Bowel Dysfunction

Radiotherapy increased the risk of bowel symptoms, whereas the frequency of bowel dysfunction after radical prostatectomy or in men on surveillance was no different from that in the men in the control group (Fig. 2).

### Health‐Related Quality of Life

Study participants were asked about sexual, urinary and bowel function separately: ‘If you were to spend the rest of your life with your condition just the way it is now, how would you feel about that?’. The proportion of men who responded ‘mostly dissatisfied’, ‘unhappy’ or ‘terrible’ regarding sexual function was 35% for all men treated for prostate cancer as compared with 18% for men in the control group. The corresponding proportions were 11 vs 10% for urinary function and 7 vs 4% for bowel function.

### Sensitivity Analyses

Patient and tumour characteristics for non‐responders were compared with responders. Non‐responders tended to be older, have more comorbidities, a lower educational level and were more often managed with surveillance and treated with hormonal therapy, although differences were small (data not shown); however, in a multivariable analysis performed in cases only including age, comorbidity, martial status and educational level (as we did not have these data for control subjects), with active surveillance as reference, the increases in risk for men in each of the active treatment groups remained similar (Fig. S1). This was true for both imputation alternatives.

We also performed separate analyses for men with low‐ and intermediate‐risk prostate cancer (Fig. S2). Overall, the risks of side effects were similar for both risk groups.

## Discussion

In this large, population‐based study in Sweden, the impact on functional outcomes remained substantial for all treatment methods more than a decade after prostate cancer diagnosis. The majority of men experienced erectile dysfunction or were sexually inactive, almost 20% reported urinary incontinence and 14% had bowel symptoms, all of which affected quality of life.

A general and consistent pattern throughout all domains (sexual, urinary and bowel function) was that men who had received multiple treatments, in particular men who received ADT had poor functional outcomes compared with men who received one single curative treatment.

### Strengths

The present study was population‐based and captured all treatment modalities provided by all types of healthcare providers in Sweden. We used self‐administered and validated questionnaires to assess the patient‐reported functional outcomes. Moreover, the present study included disease risk classification and thus enabled us to specifically study men who underwent treatment for localized prostate cancer only.

### Limitations

The present study had several limitations. Data were cross‐sectional in nature, and not longitudinal, which would have been ideal. The present study did not include a baseline assessment before treatment; however, we did have a comparison group of age‐ and county‐matched men free from prostate cancer. The risk of adverse effects were consistently and significantly higher across all domains among cases compared with controls.

The response rates were suboptimal and there was potential bias because we cannot exclude the possibility that response rate was not related to outcome measures. A descriptive analysis of non‐responders compared with responders showed some minor differences in patient and tumour characteristics (data not shown). We addressed this using multivariate imputation by chained equations, and the patterns seen in the primary analysis remained. In sensitivity analyses restricted to treated patients (with surveillance as the reference group), estimates adjusted for age as well as estimates adjusted for age, comorbidity, marital status and education were essentially the same, suggesting that these variables were not important confounders. A limitation of this study is the lack of information on the control group other than age.

The results of the present study need to be interpreted in the light of its observational design and the fact that it includes a historical cohort. Older men with more comorbid conditions and who are less fit may be more likely to undergo radiotherapy than radical prostatectomy; therefore, confounding by indication by tumour characteristics and comorbidity cannot be excluded, despite adjusting for age. Moreover, men in this study were diagnosed between 1997 and 2002 and some men received secondary treatments within 12 years. Treatment may have improved over time and contemporary outcomes may be superior.

The present study is consistent with previous intermediate‐ and long‐term (2–15 years) studies showing declines in sexual, urinary and bowel domains after prostate cancer treatment, especially erectile dysfunction 1, 7, 18. Our findings are very similar to those from the Scandinavian Prostate Cancer Group Study Number 4 (SPCG‐4) which had a median follow‐up of 12 years and median participant age of 77 years at evaluation, which reported a prevalence of erectile dysfunction of 84% after radical prostatectomy, 80% in men on watchful waiting and 46% among control subjects 19.

Men on surveillance in the present study had similar risks for all four outcomes (erectile dysfunction, urinary urgency, urinary incontinence and bowel dysfunction) compared with the men in the cancer‐free control group.

The definition and reporting of erectile function after prostate cancer treatment, mainly radical prostatectomy, is inconsistent in the literature 18. A common definition of potency is ‘erections sufficient for intercourse, with or without phosphodiesterase type 5 inhibitors’ 18. The National Institute of Health consensus conference defined erectile dysfunction as ‘the consistent inability to obtain and/or maintain an erection sufficient for satisfactory sexual performance’ 20, a definition that did not include any mention of use of medication or other treatment for erectile dysfunction; therefore, men who reported use of phosphodiesterase type 5 inhibitors were included with their reported scores, whereas men who reported use of transurethral suppositories or intracavernous injections used for erectile dysfunction were considered to have erectile dysfunction using the first definition.

The present study confirms previous observations made by Sanda et al. 1, who found a high risk of urinary incontinence 2 years after radical prostatectomy. Men who received multiple treatments had the highest risk of poor functional outcomes. This finding is consistent with data from the CaPSURE registry, in which men who received multiple treatments had a sharper decline in urinary and sexual function at 2 years compared with men who only received primary treatment 21. Furthermore, this corroborates previous findings by Sanda et al. 1 that neoadjuvant ADT before radiotherapy was associated with worse scores in multiple quality‐of‐life domains, especially urinary incontinence. These findings highlight the importance of critically assessing the need for secondary treatment and of informing men about the risks of more side effects.

In the SPCG‐4 trial, the proportion of men with urinary leakage at least once daily increased with time. At a median follow‐up of 12 years, 41% of men who had undergone prostatectomy, 11% of men on watchful waiting and 3% of men in a control group had urinary leakage 19. In the Prostate Cancer Outcomes Study (PCOS) study, only 14% of men reported frequent urinary leakage or no urinary control 5 years after prostatectomy 22. In the present study, 23% reported urinary leakage 12 years after diagnosis in all treatment groups combined. Differences in definition of urinary incontinence and follow‐up time may contribute to differences in observed estimates.

Radical prostatectomy was associated with a significantly lower risk of urinary urgency in the present study, corroborating results from previous studies that radical prostatectomy prevents age‐dependent progression of LUTS 23.

In the present study the risk of long‐term bowel dysfunction was high after radiotherapy. In the PCOS study, men treated with radiation therapy initially had more bowel urgency compared with radical prostatectomy, but there were no significant differences between the groups by 15 years 7.

As the patient population in the present study stemmed from a truly population‐based, nationwide sample, and were not preselected patients, results are likely to represent the real‐life scenario. Furthermore, outcomes assessed through questionnaires give a fair view of the patients' perspective and may protect against bias that might arise, for example, when patients minimize symptoms when asked by their physician. The present study showed a substantial risk of poor functional outcomes 12 years after the initial treatment, underlining the need for consistent improvement in the delivery of curative treatment in order to reduce the rate of adverse effects. Because the treatments were delivered more than a decade ago and by all types of healthcare providers, including low‐volume centres, our results probably represent a worst‐case scenario, as cancers currently diagnosed are more likely to be low risk and treatment delivery has improved, including greater centralization of care 24.

In conclusion, a high proportion of men treated for localized prostate cancer with curative intent experienced sexual, urinary and bowel dysfunction >12 years after treatment and men who received several treatments were at particularly high risk of poor functional outcomes. Our findings highlight the importance of careful patient selection and underline the need for a consistent improvement in the delivery of curative treatment given the potential long‐term functional implications.

## Conflict of Interest

None declared.

**Abbreviations**
OR: odds ratio
ADT: androgen deprivation therapy
CaPSURE: Cancer of the Prostate Strategic Research Endeavor
NPCR: National Prostate Cancer Register of Sweden
IQR: interquartile range
IIEF‐5: International Index of Erectile Function‐5
SPCG‐4: Scandinavian Prostate Cancer Group Study Number 4
PCOS: Prostate Cancer Outcomes Study

## Supporting information


**Fig S1.** Odds ratios and 95% confidence intervals for the risk of adverse functional outcomes by treatment, adjusted for age (imputation alternative 1) and adjusted for age, comorbidity, marital status and education (imputation alternative 2).

**Fig S2.** Odds ratios and 95% confidence intervals for the risk of adverse functional outcomes by treatment and risk group, and adjusting for age, comorbidity, marital status and education.
