# Supplementary figures and images for: Population‐based study of long‐term functional outcomes after prostate cancer treatment
Source: BJU Int. 2015 Jun 23;117(6B):E36–45. doi: 10.1111/bju.13179 (PMC4637260; doi:10.1111/bju.13179)

### Imputation alternative 1

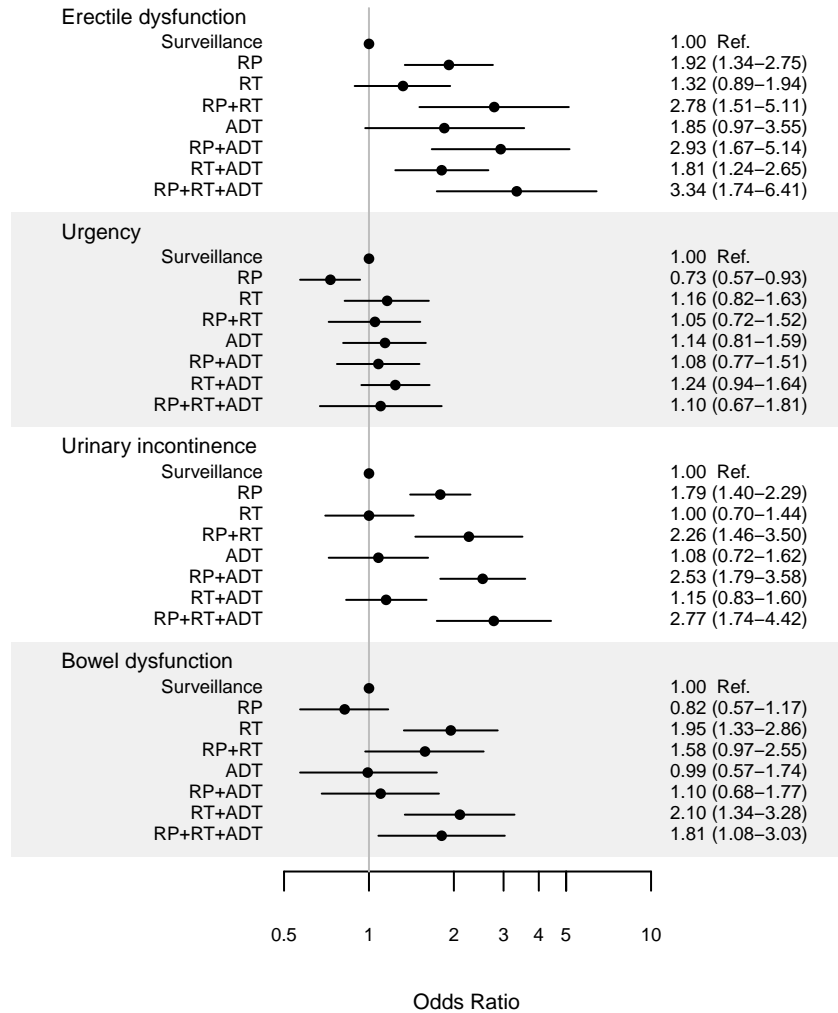

### Imputation alternative 2

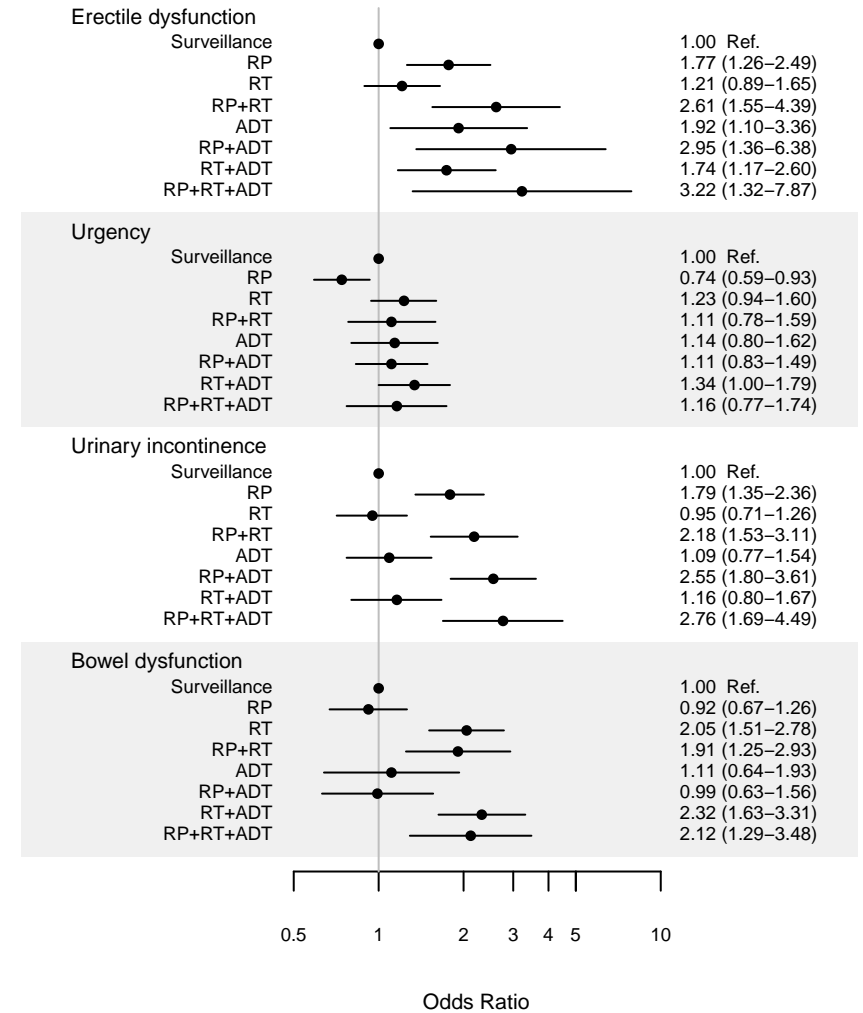

Supplement: Supplementary file 1 — Fig S1. Odds ratios and 95% confidence intervals for the risk of adverse functional outcomes by treatment, adjusted for age (imputation alternative 1) and adjusted for age, comorbidity, marital status and education (imputation alternative 2). [file BJU-117-E36-s001.pdf]

### Low risk prostate cancer

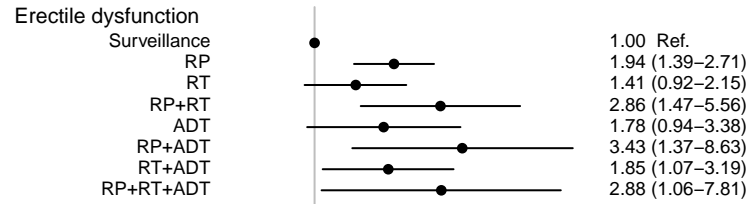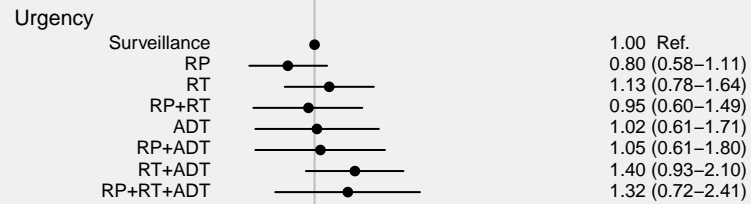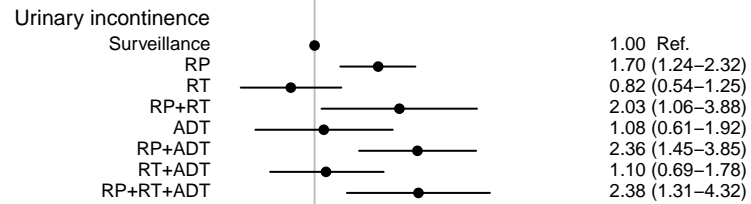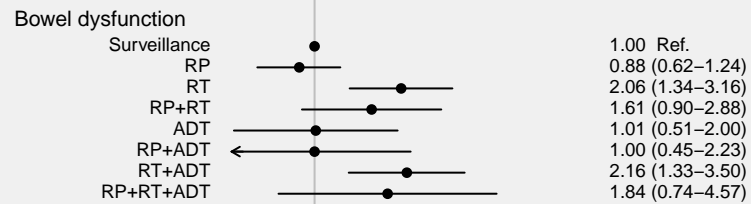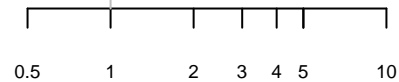

Odds Ratio

### Intermediate risk prostate cancer

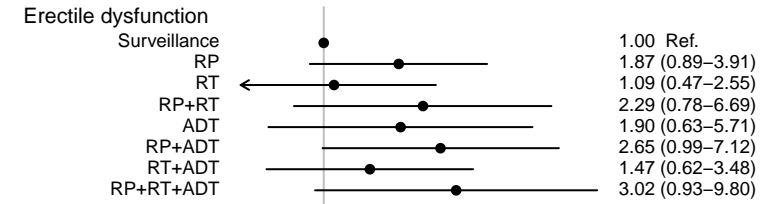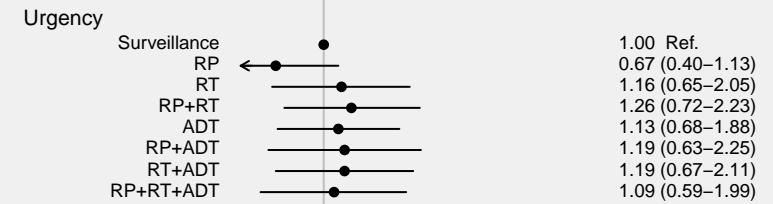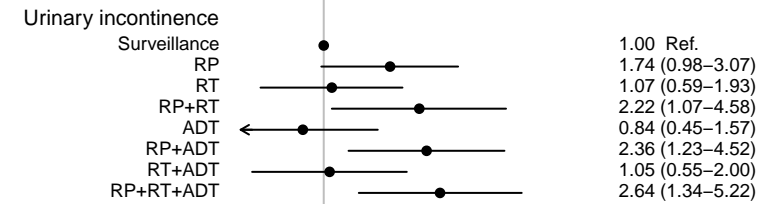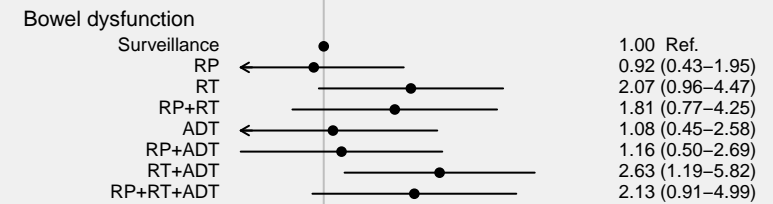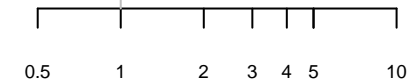

Odds Ratio

Supplement: Supplementary file 2 — Fig S2. Odds ratios and 95% confidence intervals for the risk of adverse functional outcomes by treatment and risk group, and adjusting for age, comorbidity, marital status and education. [file BJU-117-E36-s002.pdf]
